# Supplementary figures and images for: Integrating field surveys and remote sensing to optimize phosphorus resource management for rainfed rice production in the Central plateau of Burkina Faso
Source: PLoS One. 2024 Oct 25;19(10):e0312070. doi: 10.1371/journal.pone.0312070 (PMC11508118; doi:10.1371/journal.pone.0312070)

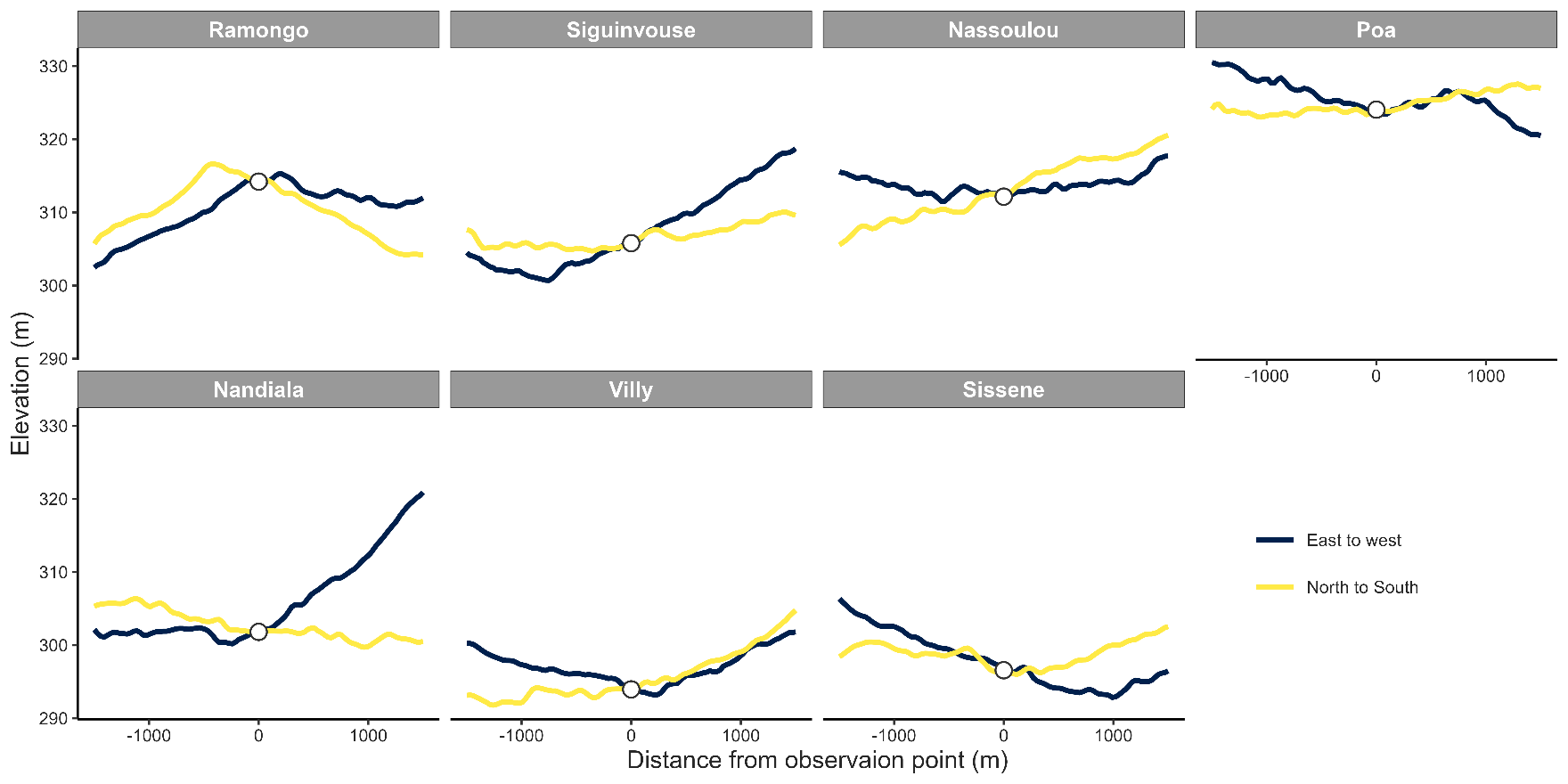


**Fig S1. Topographical characteristics of the study sites.**

Supplement: S1 Fig — (DOCX) [file pone.0312070.s001.docx]
