# Supplementary material for: Integrating field surveys and remote sensing to optimize phosphorus resource management for rainfed rice production in the Central plateau of Burkina Faso
Source: PLoS One. 2024 Oct 25;19(10):e0312070. doi: 10.1371/journal.pone.0312070 (PMC11508118; doi:10.1371/journal.pone.0312070)
Supplement: S2 Fig — Photographs of the soil profiles (upper panel) and soil texture distributions (lower panel). Horizon names were set following the World Reference Base for Soil Resources [30]. (DOCX) [file pone.0312070.s002.docx]

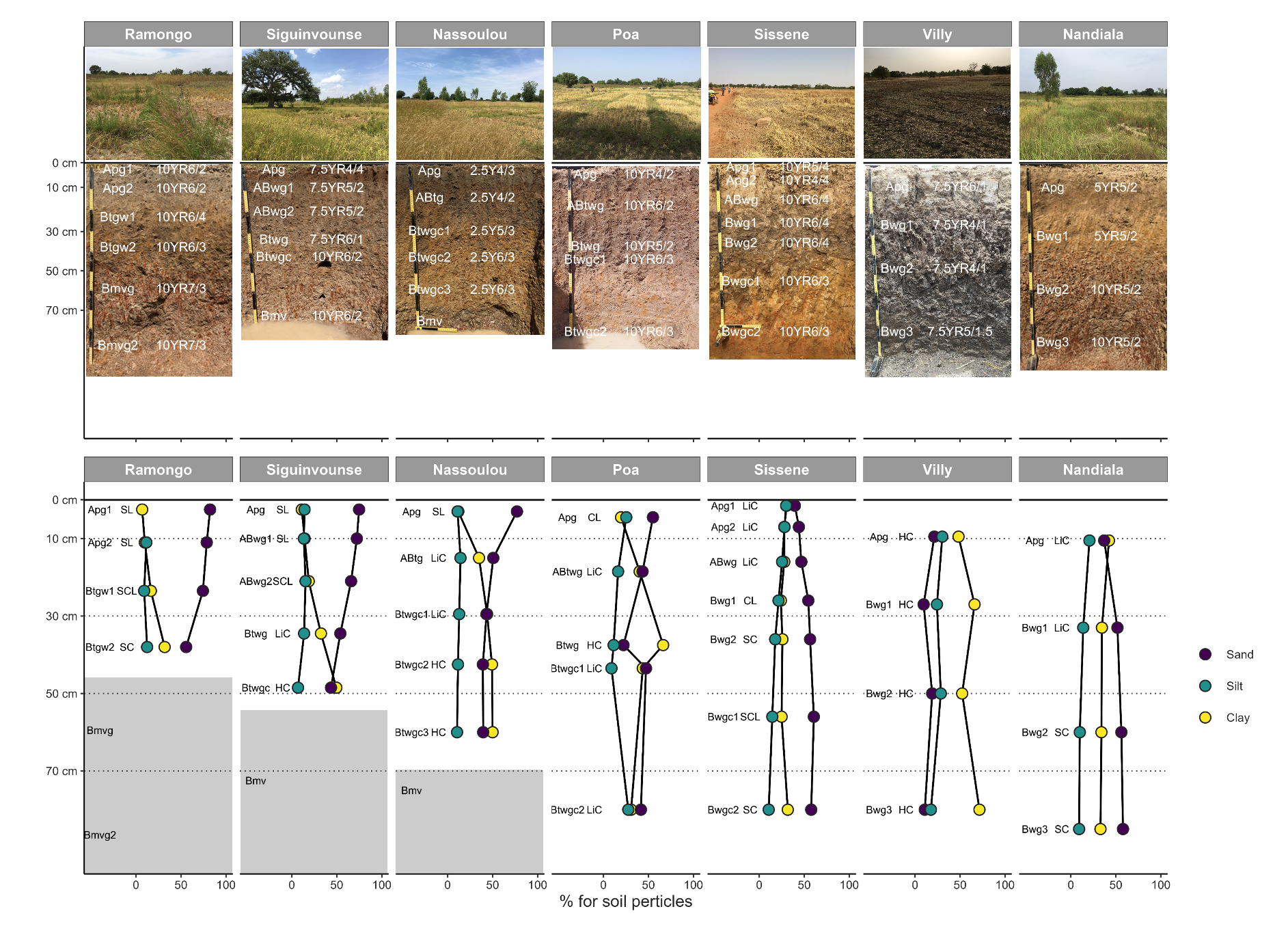


**Fig S2. Photographs of the soil profiles (upper panel) and soil texture distributions (lower panel).** Horizon names were set following the World Reference Base for Soil Resources [30].
