# Supplementary material for: Integrating field surveys and remote sensing to optimize phosphorus resource management for rainfed rice production in the Central plateau of Burkina Faso
Source: PLoS One. 2024 Oct 25;19(10):e0312070. doi: 10.1371/journal.pone.0312070 (PMC11508118; doi:10.1371/journal.pone.0312070)
Supplement: S1 Table — Mottling is described in the order of abundance, contrast, shape, and species. The mottled color is expressed in parentheses. Abbreviations: N, None; V, Very few (0–2%); F, few (2–5%); C, Common (5–15%); M, Many (15–40%); A, Abundant (>40%). Contrast: F, Faint; D, Distinct; P, Prominent. Shape: RO, Root-like; FI, Filmy; IR, Irregular; SP, Speckled; CL, Cloudy. Species: Fe, Iron; Mn, Manganese. Concretions are described based on abundance and species. The abbreviations are the same as those used for mottling. The concentrations of Fe2+ ions were related to the strength of the reaction with the 0.2% α-dipyridyl solution in 10% acetic acid. Strength of reaction:–, none; ±, very weak; +, weak; ++, strong; +++, very strong. Rocks are described in the order of abundance, weathering state, size, and shape. Abbreviations: N, None; F, Few (0–5%); C, Common (5–10%); M, Many (10–20%); A, Abundant (20–50%); D, Dominant (>50%). State of weathering: F, Fresh; SL, Slightly weathered; W, Weathered; ST, Strongly weathered. Size: FC, Fine gravel (0.2–1 cm); G, Gravel (1–5 cm); S, Stone (5–10 cm); LS, Large stone (10–20 cm); UN Unknown. The structure of the rocks is described in the order of their grades, sizes, and types. Grade: W, Weak; M, Moderate; S, Strong. Size: VF, Very fine (<5 mm); F, Fine (5–10 mm); M, Medium (10–20 mm); C, Coarse (20–50 mm); VC, Very coarse (>50 mm). Type: SB, Subangular block; PL, Platy; M, Massive; N, None. (DOCX) [file pone.0312070.s003.docx]

**Table S1. Soil morphological properties based on the World Reference Base for Soil Resources [30].**

| Site | Horizon | Depth | Color | Mottling | Concretions | Fe^2+^ | Rocks | Structure | Hardness |
| --- | --- | --- | --- | --- | --- | --- | --- | --- | --- |
| Soil type |  | (cm) |  |  |  |  |  |  |  |
| Nassoulou | Apg | 0-6 | 10YR6/2 | C, F, RO, Fe; C, F, CL, Fe (10YR6/8) | N | - | N | W, VF-M, SB | - |
| Abruptic Relictigleyic Luvisols | ABtg | 6-24 | 10YR6/2 | C, F, RO, Fe; C, F, CL, Fe (10YR6/8) | N | - | N | W, VF-M, SB | 17 |
| (LV-rl.ap) | Btwgc1 | 24-35 | 10YR6/4 | M, F, RO, Fe; M, F, CL, Fe (10YR6/8) | N | - | N | W, VF-M, SB | 30 |
|  | Btwgc2 | 35-50 | 10YR6/3 | A, D, FL, Fe (7.5YR6/8) | N | - | N | W, VF-C, SB | 29 |
|  | Btwgc3 | 50-70 | 10YR7/3 | A, P, FL, Fe (2.5YR4/8) | N | - | A, ST, UN | W, VF-C, SB | 29 |
|  | Bmv | 70-80+ | 10YR7/3 | A, P, FL, Fe (2.5YR4/8) | VF, Mn | - | A, ST, UN | W, VF-C, SB | 28 |
| Siguinvousse | Apg | 0-5 | 7.5YR4/4 | C, D, RO, Fe; C, D, CL, Fe (5YR4/8) | N | ++ | N | N | 28 |
| Ferric Relictigleyic Lixisols | ABwg1 | 5-15 | 7.5YR5/2 | M, P, RO, Fe; M, P, CL, Fe (5YR4/8) | N | - | N | M, F-M, SB | 28 |
| (LX-rl.fr) | ABwg2 | 15-27 | 7.5YR5/2 | M, P, RO, Fe; M, P, CL, Fe (5YR5/8) | N | - | N | W, F-M, SB | 23 |
|  | Btwg | 27-42 | 7.5YR6/1 | F, P, RO, Fe; M, P, CL, Fe (7.5YR5/6) | N | - | N | W, F, SB | 30 |
|  | Btwgc | 42-55 | 10YR6/2 | A, P, FL, Fe (2.5YR4/8) | N | - | C, ST, UN | W, M-C, SB | 33 |
|  | Bmv | 55-90+ | 10YR6/2 | A, P, FL, Fe (2.5YR4/8) | N | - | A, ST, UN | W, M-C, SB | 33 |
| Ramongo | Apg1 | 0-5 | 2.5Y4/3 | VF, P, RO, Fe (5YR4/8) | N | +++ | N | N | 27 |
| Ferric Albic Relictigleyic Lixisols | Apg2 | 5-17 | 2.5Y4/2 | M, P, RO, Fe (5YR4/8) | N | ++ | N | W, M-C, SB | 24 |
| (LX-rl.fr) | Btgw1 | 17-30 | 2.5Y5/3 | F, P, RO, Fe (7.5YR5/8); A, P, CL, Fe (10YR6/6) | N | - | F, F, G | W, M-C, SB | 25 |
|  | Btgw2 | 30-46 | 2.5Y6/3 | A, D, IR, Fe (10YR5/6) | N | - | F, F, FC; C, SL, FC | W, M-C, SB | 25 |
|  | Bmvg | 46-73 | 2.5Y6/3 | A, D, IR, Fe (10YR5/6) | N | - | C, F-SL, FC | W, F, SB | 13 |
|  | Bmvg2 | 73-100+ |  |  |  | + | D, F, FC-G; F, F, LS | N | 16 |
| Nandiala | Apg | 0-21 | 10YR4/2 | C, P, RO, Fe; M, P, CL, Fe (7.5YR5/6) | N | ± | N | W, F, SB | 18 |
| Eutric Relictigleyic Cambisols | Bwg1 | 21-45 | 10YR6/2 | F, P, RO, Fe; M, P, CL, Fe (7.5YR5/6) | N | - | N | M, VF-F, SB | 14 |
| (CM-rl.eu) | Bwg2 | 45-75 | 10YR5/2 | C, P, FL, Fe (7.5YR5/6) | N | - | N | W, VF-F, SB | 17 |
|  | Bwg3 | 75- | 10YR6/3 | A, P, FL, Fe (7.5YR5/6) | N | + | F, ST, FC | W-M, VF-F, SB | 16 |
| Villy | Apg | 0-19 | 10YR6/3 | A, P, FL, Fe (7.5YR5/6) | N | ± | F, ST, FC | W-M, VF-F, SB | 16 |
| Eutric Sideralic Relictigleyic Cambisols | Bwg1 | 19-35 | 10YR5/4 | F, D, RO, Fe (10YR5/8) | N | ± | N | W, F-C, SB; W, M, PL | 20 |
| (CM-rl.se.eu) | Bwg2 | 35-65 | 10YR4/4 | C, D, RO, Fe (10YR5/8) | N | ± | N | W, F-C, SB | 23 |
|  | Bwg3 | 65- | 10YR6/4 | C, D, RO, Fe (10YR5/8) | N | ± | N | W, F-C, SB | 24 |
| Poa | Apg | 0-9 | 10YR6/4 | M, D, RO, Fe; M, D, CL, Fe (10YR5/8) | N | ± | N | W, F-M, SB | 21 |
| Abruptic Relictigleyic Lixisols | ABtwg | 9-28 | 10YR6/4 | M, P, RO, Fe; M, P, CL, Fe (10YR6/6, 7.5YR5/8) | N | - | N | W, F-C, SB | 22 |
| (LX-rl.ap) | Btwg | 28-47 | 10YR6/3 | M, P, IR, Fe (5YR3/6) | VF, Mn | - | F, ST, G | M, F-C, SB | 12 |
|  | Btwgc1 | 47-60 | 10YR6/3 | A, P, IR, Fe (5YR3/6) | F, Fe; VF, Mn | - | F, ST, G | M, F-C, SB | 31 |
|  | Btwgc2 | 60-100+ | 5YR5/2 | M, D, RO, Fe (5YR/8) | VF, Fe | - | N | S, C, SB | 14 |
| Sissene | Apg1 | 0-3 | 5YR5/2 | F, D, RO, Fe; F, D, CL, Fe (5YR6/8, 10YR6/8) | VF, Fe | - | N | S, C, SB | 17 |
| Eutric Sideralic Relictigleyic Cambisols | Apg2 | 3-11 | 10YR5/2 | A, D, IR, Fe (5YR5/8) | F, Fe | - | N | S, M, SB | 22 |
| (CM-rl.se.eu) | ABwg | 11-21 | 10YR5/2 | A, D, IR, Fe (5YR4/8, 10YR5/8) | N | - | N | W, M, SB | 22 |
|  | Bwg1 | 21-31 | 7.5YR6/1 | M, F, RO, Fe (7.5YR6/8) | N | - | N | S, VC, SB | 24 |
|  | Bwg2 | 31-41 | 7.5YR4/1 | F, F, RO, Fe; F, F, FL, Fe (7.5YR6/8); M, F, RO, Mn (2/0) | N | - | N | M, M-C, SB | 19 |
|  | Bwgc1 | 41-71 | 7.5YR4/1 | F, F, IR, Fe (7.5YR6/8); F, F, SP, Mn (2/0) | N | - | N | M, VC, SB | 31 |
|  | Bwgc2 | 71- | 7.5YR5/1.5 | C, D, IR, Fe (5YR5/8); VF, F, SP, Mn (2/0) | N | - | N | W, C, SB | 25 |

Mottling is described in the order of abundance, contrast, shape, and species. The mottled color is expressed in parentheses. Abbreviations: N, None; V, Very few (0–2%); F, few (2–5%); C, Common (5–15%); M, Many (15–40%); A, Abundant (>40%). Contrast: F, Faint; D, Distinct; P, Prominent. Shape: RO, Root-like; FI, Filmy; IR, Irregular; SP, Speckled; CL, Cloudy. Species: Fe, Iron; Mn, Manganese. Concretions are described based on abundance and species. The abbreviations are the same as those used for mottling. The concentrations of Fe2+ ions were related to the strength of the reaction with the 0.2% *α*-dipyridyl solution in 10% acetic acid. Strength of reaction: –, none; ±, very weak; +, weak; ++, strong; +++, very strong. Rocks are described in the order of abundance, weathering state, size, and shape. Abbreviations: N, None; F, Few (0–5%); C, Common (5–10%); M, Many (10–20%); A, Abundant (20–50%); D, Dominant (>50%). State of weathering: F, Fresh; SL, Slightly weathered; W, Weathered; ST, Strongly weathered. Size: FC, Fine gravel (0.2–1 cm); G, Gravel (1–5 cm); S, Stone (5–10 cm); LS, Large stone (10–20 cm); UN Unknown. The structure of the rocks is described in the order of their grades, sizes, and types. Grade: W, Weak; M, Moderate; S, Strong. Size: VF, Very fine (<5 mm); F, Fine (5–10 mm); M, Medium (10–20 mm); C, Coarse (20–50 mm); VC, Very coarse (>50 mm). Type: SB, Subangular block; PL, Platy; M, Massive; N, None.
