# Supplementary material for: Integrating field surveys and remote sensing to optimize phosphorus resource management for rainfed rice production in the Central plateau of Burkina Faso
Source: PLoS One. 2024 Oct 25;19(10):e0312070. doi: 10.1371/journal.pone.0312070 (PMC11508118; doi:10.1371/journal.pone.0312070)
Supplement: Attachment — Submitted filename: Response to reviwer.docx [file pone.0312070.s004.docx]

To Reviewer 1

Thank you very much for your constructive and accurate comments on our manuscript.

We have revised the manuscript according to your comments.

The major changes made are outlined below:

1. **Style Revision**: We have modified the style throughout the manuscript to align with the journal's guidelines.
2. **Selection Method and Research Permissions**: We have revised the sections concerning the selection method of the research site and obtaining permissions for the research to ensure clarity and compliance.
3. **Limitations and Future Prospects**: We have included a section discussing the limitations of our study and outlining potential avenues for future research.

Reviewer #1:

C: According to the authors, the study aimed to identify the soil type distribution and factors impacting the P fertilization effect on lowland rice production by integrating a field survey and an estimation of soil water conditions using a remote sensing approach. The topic is very important as in most of the soils of sub-Saharan Africa, crop production is limited by the low P availability of soils. Moreover, knowing the factors influencing phosphate fertilizers efficiency will allow a site-specific recommendation of fertilizer types and amount and then maximize crop production.

However, the objective of the work must be reformulated to show the relevance of the study. In fact, through the manuscript, the work is about to evaluate the water condition, mainly inundation score of rice cultivation area and its useability in recommending phosphorus fertilizers for rice production. Moreover, previous studies in the studied area have highlighted the relationship between soil chemical and physical characteristics and the response of crops to phosphorus fertilizers (Nakamura et al., 2020, Fukuda et al., 2021, Iwasaki et al., 2021; Soma et al., 2023).

A: Thank you for pointing this out. I think you are exactly right.

We have revised the following (L101-104)

Therefore, this study aims to evaluate the water condition of rice cultivation area and its useability in recommending P fertilizers for rice production. Achieving these objectives could contribute to increasing rice productivity in the upper reaches of rivers in Burkina Faso and SSA

C:-Line 122 Materials and methods: define the expression “local communities” and explain why you selected farmers by “local communities”.
- Materials and methods: Is one farmer per village for a total of 7 villages representative of the studied area? How the authors expect to manage phosphorus fertilization using inundation score in some area such as Nandiala, as some time this location was drop during data analysis?

C: - Results: There was not a significant relationship between inundation score and yield in soil receiving P fertilizers. Can we still use this parameter to map P fertilization? More explanations needed.

A： Thank you for bringing this to our attention. We have incorporated the following revisions in response to the editor's comments.

During the site selection process, it was imperative to choose plots with varying topography and water environments, spanning from upstream to downstream of the watershed. Subsequently, we obtained survey permissions from the farmers through INERA. While at one site (Nandiala), where man-made impacts were evident, the inundation score estimated from the GIS proved inadequate, at other sites, it was estimated with a high degree of accuracy. These findings highlight the limitations of our study and underscore areas for future improvement.

Please see below:

L107-113

L476-481
