# Supplementary material for: Integrating field surveys and remote sensing to optimize phosphorus resource management for rainfed rice production in the Central plateau of Burkina Faso
Source: PLoS One. 2024 Oct 25;19(10):e0312070. doi: 10.1371/journal.pone.0312070 (PMC11508118; doi:10.1371/journal.pone.0312070)
Supplement: Attachment — Submitted filename: Response to Reviewers.docx [file pone.0312070.s005.docx]

**Response to Reviewers**

| Reviewers  We greatly appreciate the effort and time reviewers and editors put into improving our manuscript. If any responses are unclear or you wish to make additional changes, please let us know.  Respectfully yours,  Shinya IWASAKI (Corresponding author) | |
| --- | --- |
| Reviewer #2: | Line No. for change |
| C: The manuscript aims to investigate the factors influencing the efficiency of rock phosphate application on rainfed lowland rice production in Burkina Faso and to generalize the findings using remote sensing data. The topic addressed in this article is worthy of investigation, given the importance of finding alternatives to the costly mineral fertilizers currently in use. I also acknowledge the efforts invested in identifying the soil type in the seven fields. However, there are some questionable conclusions, flaws in the writing, and missing information that I believe should be addressed to improve the scientific quality of the paper. Therefore, I recommend revising the paper by providing a more coherent interpretation and including the missing information. While I apologize for providing so many remarks at this stage of the review process, please remember that my sole objective is to improve your paper.  A: We sincerely appreciate the very detailed points you have pointed out and the valuable time you have given us. Please see below for the corrections to each comment. |  |
| •C: Materials and methods of Lines 150-152 Why did you cite Nakamura et al. (2020)? I do not perceive the link. Are these data already analyzed and published? What are the differences with the current dataset?  A: Thank you so much for your comment. Actually, the row data set was already published by Nakamura et al., 2020. However, we believe that it is worth re-analyzed by combining soil profile surveys and remote sensing. We revised the relevant section by adding the explanation. | L143-148 |
| C: Did you check the conditions for the application of Pearson’s correlation coefficient? In case the conditions are not met, Spearman’s correlation is an alternative.  A: Thank you so much for your crucial point; we checked the normality and judged that Spearman’s correlation is appropriate. So Table 2 was updated. | L196-198  Table 2 |
| C: Discussion: To corroborate your claim that P is the most important limiting factor in these sites, I suggest in the discussion to compare the N, P and K values to average values or threshold of soil fertility in rainfed lowland rice fields in sub- Saharan Africa.  A: We also agree with this point. At the seven sites included in this study, the available P (Bray1-P) was substantially lower than the threshold value of 7 to 9 (mg P kg^−1^) at all sites, indicating that P is likely the limiting factor. However, the increased yield at the NK site compared to the CT site and the consideration that in previous reports, PK is considered to be more limiting due to the interaction of NPK rather than a lack of PK alone were taken into account. Therefore, we revised the discussion with relevant citations. | L430-445 |
| C: The pages are not numbered.  A: Page number was added |  |
| Line 28: Were the duration of submergence and cumulative water level recorded or extracted from remote sensing data?  A: It was recorded in the field manually. We added the information. | L28 |
| Lines 30-32: I do not see the relevance of this paragraph in the Abstract, as these are not key points of this study.  Besides, no research objective mentioned in the Abstract is linked to this result. I suggest deleting these sentences  A: We agree with you about deleting the sentence. | Abstaract |
| • Lines 33-34: What do you mean by “water environment”? Are you referring to soil water conditions? Please use the appropriate expression.  A: Thank you for your suggestion; the word water environment has changed to soil water condition thought the manuscript. |  |
| • Lines 81-82: Is this information on the yield of sorghum relevant for this study?  • Lines 82-84: Is this insight on cowpea relevant to this study? Why not look for studies on rice in West Africa?  A: Thank you for your suggestion. These references to sorghum and cowpea were removed. Instead, a reference showing the soil type effect on rain-fed lowland rice cultivation was added. | L67-71 |
| • Lines 87-88: Is the French translation necessary? What do you mean by "lowland riverine areas"? This sounds a bit awkward. You could just use "rainfed lowland rice."  A: Thank you for your suggestion. It was changed to “rainfed lowland rice cultivation areas” | L82-84 |
| ・Line 108: I wonder if these are cultivation areas of just villages.  ・Line 111: I wonder if these are cultivation areas of just villages.  A: Yes, it is just the village name. We modified the sentence to simplify. | L96-100 |
| • Line 113: It seems that the word “pattern” is missing.  A: It was added | L103 |
| • Line 124: Here, you are talking about soil particle size proportion  A: Yes, thank you for the indication | L115 |
| How did you determine the soil texture? Which textural triangle did you use (see also Table 1)?  A: Soil texture class was determined according to WRB. A relevant sentence was added. | L119 |
| • Line 161-162: Please provide more understandable labels for each treatment. Please be clear  A: Thank you for the indication. We rethought the necessity of the sentence and judge to delete it. |  |
| • Line 169: There is redundancy. Please delete “in a planting space”.  A: It was deleted. | L159 |
| • Lines 222: I suggest writing “Table S1” and “Fig. S1”  A: I’m sorry for the careless mistake, it was changed to Table S1” and “Fig. S1 | L218 |
| • Line 229: cmolc. It better to avec “c “as an indice.  A: I’m sorry for the careless mistake; it was changed through the manuscript. |  |
| • Could you please spell out Bmvg, Btwgc, and Bmv?  A: I tried, but it is difficult because these are not simple abbreviations but soil terms with a lump meaning. And it is not spelled out generally. Therefore, I added the reference. Thank you for your understanding. |  |
| • Lines 247-248: Please rephrase. Please avoid interpreting a correlation as a cause-effect relationship.  A: Thank you for your indication, I agree. The sentence was rephrased. | L244-245 |
| • Lines272: NK + TSP > NK + PR. This is not statistically different at p < 0.05. Therefore, is writing this true?  A: I revised the sentence to justify. | L267-270 |
| • Line 274: Please rephrase the title of Table 3  A: It was changed to “Table 3 Soil water conditions and rice grain yield under different fertilizer treatments.” | Table 3 |
| • Line 286: six or five? 7-2 = 6 or 5?  A: I’m sorry for the mistake; five is correct. | L288 |
| • Lines 290-292: “Fig 3. Relationships between (a) water accumulation and longitudinal convexity and (b); water accumulation and cross-sectional convexity.  A: Thank you for your kind suggestion. It was revised | L293-295 |
| • Lines 301-302: Please rephrase. It does not make sense when expressed like this.  A: Thank you for the indication; it was rephrased. | L303-307 |
| • Line 336: “positive” relationships?  A: Yes, it is positive. Thank you so much. | L340 |
| • Line 349: One decimal could be enough.  A: Thank you so much for your suggestion. I agree. | Figure 6 |
| • Lines 428-431: The focus of this study is rainfed lowland rice, correct? If so, I recommend comparing your average yield with those specific to the rainfed lowland rice production system. The average yield from USDA (2018) includes irrigated lowland, rainfed lowland, and rainfed upland, making the comparison unfair.  A: Thank you for the important point. Introduction and relevant discussion was revised to use average rice grain yield under rainfed condition. | L50-54  L446-448 |
| • Lines 436: What do you mean by “water environment”? Are you referring to soil water conditions? Please use the appropriate expression.  A: Thank you for your suggestion; the word water environment has changed to soil water condition throughout the manuscript. |  |
| • Lines 455-458: Why do make this conclusion?  A: Agree that it is meaningless. It was deleted. |  |
| • Line 465: Please rephrase this sentence. Do you mean the relative grain yield of NK + PR over NK +TSP  A: Yes, I revised the sentence and y-axis of figure 6 | L356-357  Figure 6 |
| • Lines 466-471: The link and interpretation are unclear, making it difficult to grasp the explanation.  A: Thank you. After the consideration. I think the first sentence is not necessary. |  |
| • Lines 472: What do you mean by “tentative”?  A: I’m sorry, it should be “alternative” | L483, L486 |
| • Table 1: For the pH, One decimal is enough.  • Table 1: Sand (%), Clay (%)  A: Thank you so much. It was revised with other units. | Table 1 |
| • Table 2: Relationships between soil particle size proportion and physicochemical properties (n = 34). How did you obtain this sample size of 34?  This correlation include all data sets presented in Table1. | Table2 |
| • Line 476: What is the reference 49?  A: I’m sorry, it is not necessary. |  |
| • Lines 476-477: The sentence “In this study, … analysis” could be deleted. I could not perceive his relevance in this paragraph.  A: Thank you so much. It was deleted. |  |
| Line 480-481 “ .. verification results at many more sites can be expected”. What do you mean? Please rephrase this sentence.  A: The sentence was revised as “Therefore, it is desirable to increase the number of study sites and validate the results obtained in this study” | L492-493 |
| • Lines 495: What do you mean by “water environment”? Are you referring to soil water conditions? Please use the appropriate expression.  A: A: Thank you for your suggestion; the word water environment has changed to soil water condition throughout the manuscript. |  |
| • Figure 6: Please be consistent with the abbreviation. I would suggest NK + PR and NK + TSP  • Figure 6: Please correct the y-axis of Fig 6b. Do you mean the relative grain yield of NK + PR over NK +TSP?  A; Thank you for the suggestion. I employed your suggestion thought the amanuscript. |  |
