# Supplementary material for: Integrating field surveys and remote sensing to optimize phosphorus resource management for rainfed rice production in the Central plateau of Burkina Faso
Source: PLoS One. 2024 Oct 25;19(10):e0312070. doi: 10.1371/journal.pone.0312070 (PMC11508118; doi:10.1371/journal.pone.0312070)
Supplement: Attachment — Submitted filename: Response to Reviewers.docx [file pone.0312070.s006.docx]

**Response to Reviewers**

| Reviewers  We greatly appreciate the effort and time reviewers and editors put into improving our manuscript. If any responses are unclear or you wish to make additional changes, please let us know.  Respectfully yours,  Shinya IWASAKI (Corresponding author) | |
| --- | --- |
| Reviewer #2: | Line No. for change |
| C: 1. Overall impression of the revision  The authors have provided satisfactory responses and carefully revised the manuscript. I am in favor of its publication. However, there are still some minor phrasing issues and inconsistencies. As I was not able to carefully read the entire document again, I strongly suggest that the authors carefully proofread it before the final publication.  A: We appreciate your valuable time and comments. All of the authors have reviewed the text and confirmed the structure of the text and standardized the terminology and abbreviations. |  |
| •1.1. Minor issues  I suggest either specifying which data were already published in Nakamura et al. (2020) or removing this sentence altogether.  A: Thank you for your suggestion, we judged to delete the sentence. |  |
| C: • Lines 356-357: Please ensure that "NK+PR" and "NK+TSP" are used consistently throughout the document. Replace any variations or previous abbreviations with these terms.  A: We checked thought the manuscript and "NK+PR" and "NK+TSP" |  |
| C: Discussion: To corroborate your claim that P is the most important limiting fact | L429-431 |
| C: • Lines 433-435: The phrase should be revised to clarify that it is the phosphorus (P) content in the soil that is low, not the soil itself.  A: The sentence has been revised to remove ambiguity. |  |
| • Lines 443-445: The sentence is difficult to understand. Rephrasing it in a simpler way could be helpful.  A: The sentence was revised to clarify. | L439-441 |
